# State selective fragmentation of doubly ionized sulphur dioxide

**DOI:** 10.1038/s41598-021-96405-5

**Published:** 2021-08-24

**Authors:** M. Jarraya, M. Wallner, G. Nyman, S. Ben Yaghlane, M. Hochlaf, J. H. D. Eland, R. Feifel

**Affiliations:** 1 Université Gustave Eiffel, COSYS/LISIS, 5 Bd Descartes, 77454 Champs sur Marne, France; 2 Laboratoire de Spectroscopie Atomique, Moléculaire et Applications – LSAMA, Faculté des Sciences de Tunis, Université de Tunis El Manar, 2092 Tunis, Tunisia; 3 Department of Physics, University of Gothenburg, 412 58 Gothenburg, Sweden; 4 Department of Chemistry and Molecular Biology, University of Gothenburg, 405 30 Gothenburg, Sweden; 5 Department of Chemistry, Physical and Theoretical Chemistry Laboratory, Oxford University, South Parks Road, Oxford, OX1 3QZ UK

**Keywords:** Physics, Atomic and molecular physics, Atomic and molecular interactions with photons

## Abstract

Using multi-electron–ion coincidence measurements combined with high level calculations, we show that double ionisation of SO_2_ at 40.81 eV can be state selective. It leads to high energy products, in good yield, via a newly identified mechanism, which is likely to apply widely to multiple ionisation by almost all impact processes.

## Introduction

Since their detection almost a century ago by mass spectrometry, doubly charged molecular ions have attracted both experimentalists and theoreticians to investigate their formation, stability and reactivity. Their stability is a balance between Coulombic repulsion and chemical bonding, and small polyatomic dications (AB^2+^) are all unstable or metastable since the charge separation channel (A^+^ + B^+^) is located in energy well below the charge retaining channels (AB^2+^ or A + B^2+^)^1,2^. Upon fragmentation, such dications are expected to produce the low energy fragments (A^+^ + B^+^) predominantly. All fragmentations tend to produce the lowest energy accessible products, but recent experimental and theoretical studies have shown that state-to-state fragmentations of small neutral molecules may lead to unexpected results. For instance, in some VUV photodissociation of CO_2_ there is no formation of ground state CO + O species. Instead the O photoproduct is formed in the ^1^D or the ^1^S excited states^3^, whose dipole forbidden emissions are prominent features in the visible spectra of terrestrial atmospheres^4^. Similarly, the VUV photodissociation of N_2_ by ns or fs lasers leads to at least one excited atomic nitrogen instead of two ^4^S ground state nitrogen atoms^5,6^. Very recently, Zhou et al. showed that the UV photolysis of H_2_S produces excited S(^1^D) atoms (+ H_2_) instead of ground state S(^3^P)^7^. Fragmentation of even quite large singly charged molecular ions can also be state-selective rather than statistical, of which a classical example is the dissociation of C_2_F_6_^+^^8^. These state specific processes are important to planetary atmospheres, plasmas and photochemistry at the fs and shorter time scales. By contrast, no such unexpected behavior has been reported hitherto for molecular dications.

As prototypes of bond-breaking process in molecular dications, the SO_2_^2+^ spectra and dissociations induced by photoionization or electron impact have been studied for many years accompanied by molecular structure calculations at different levels of theory. These works are reviewed in Ref.^9^, which presents a resolved spectrum of the doubly-charged ions as obtained by the TOF-PEPECO method, and interpreted using high-level molecular structure theory. Also, double ionization of the SO_2_ molecule has continued to attract attention^10,11^, partly because of the great importance of the molecule in atmospheric and astrophysical contexts^12,13^. Indeed, SO_2_ is common in volcanic and biological emissions on Earth^14,15^ and it is a crucial chemical compound in sulphur physical chemistry in Earth^13^, Venus^16^, Io^17,18^ and exoplanet terrestrial atmospheres, where its photochemistry leads to the formation of sulphuric acid or sulphuric acid aerosols with well-known deleterious effects such as acid rains^19^.

From existing studies, we know that double ionization of SO_2_ at energies below the triple ionization limit leads to dissociation in four principal ways: (1) SO_2_^2+^ → O^+^ + SO^+^ or (2) SO_2_^2+^ → O^+^ + S^+^ + O or (3) SO_2_^2+^ → O_2_^+^ + S^+^or (4) SO_2_^2+^ → O + SO^2+^. A fifth pathway producing two O^+^ ions with neutral S is detectable but has extremely low intensity. Dissociative double ionization is brought about not only by direct vertical double ionization, but also by an indirect process shown up clearly in PEPECO studies of this and other small molecules^20^ by the signatures of distinct electron energies from autoionization of super-excited O, N and other atoms^21^. The well documented indirect process can be represented for SO_2_ as: SO_2_ + hv → SO_2_^+^* + e_1_^−^; SO_2_^+^* → SO^+^ + O*: O* → O^+^ + e_2_^−^.

We here report multi-particle coincidence measurements using the TOF-PEPEPIPICO technique (cf. SI). This work makes the first direct connection between the energies and states of nascent SO_2_^2+^ ion with fragmentation leading either to charge separation (SO^+^ + O^+^) or to the thermodynamically unfavoured charge retaining (SO^2+^ + O) products of O–SO bond breaking. A vital characteristic of our technique is that both ions and electrons from ionization at a point-like source are collected and detected with high overall efficiency, being uniquely capable to extract complete mass spectra from single and double ionization separately. This is done by requiring the coincident detection of a single photoelectron of appropriate (relatively high) energy in the one case, and coincident detection of an electron pair within the right energy range in the other. As references—single and double ionization mass spectra of SO_2_ photoionized at 40.8 eV are shown in the SI. These spectra confirm that the most abundant products at this energy are O^+^ + SO^+^ and that SO^2+^ is the only doubly-charged ion formed.

**Figure 1:**
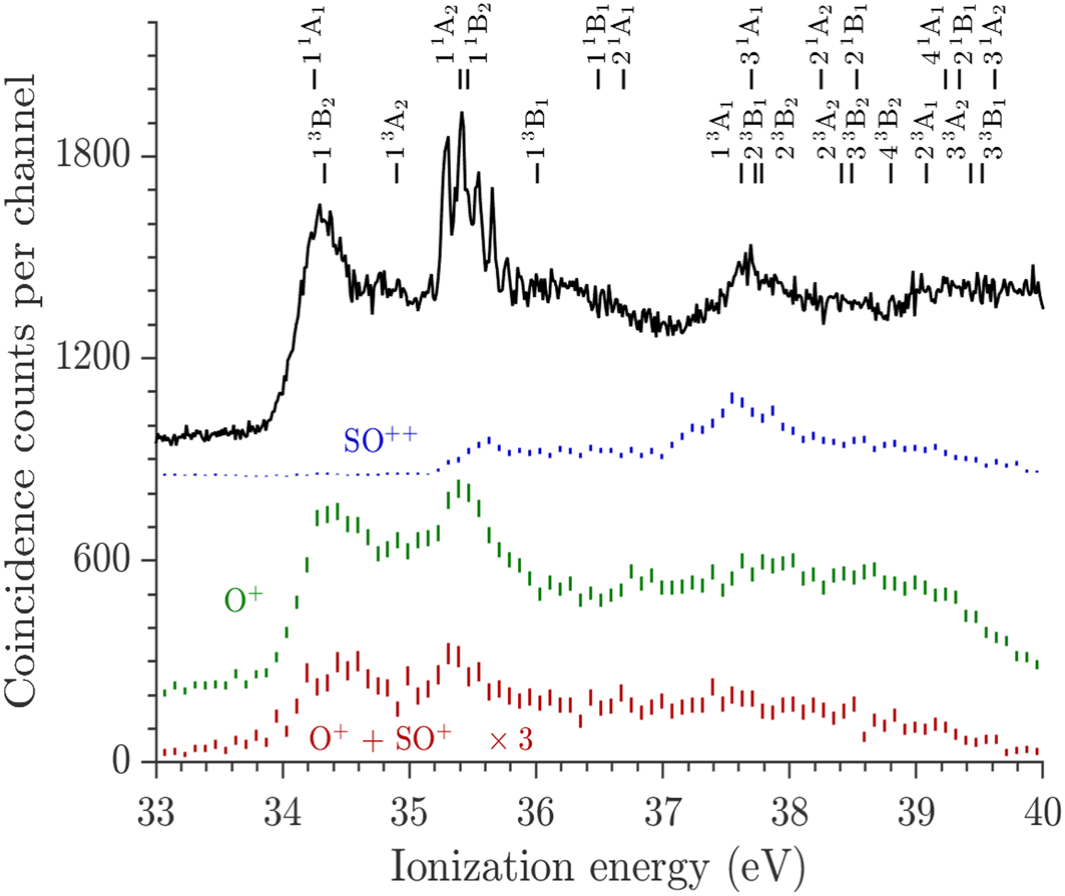
Spectra of electron pairs coincident with O^+^ + SO^+^ ion pairs and with specified single ions, are compared with a well-resolved spectrum of all electron pairs, all after photoionization of SO_2_ at the photon energy hν = 40.81 eV. The SO^2+^ and O^+^ curves are displaced vertically on a common scale (with constant off-sets) and show true relative intensities, while the other spectra included for comparison have intensity scales adjusted for display. The combs correspond to the MRCI/aug-cc-pV(Q + d)Z computed vertical double ionization energies of SO_2_ given in Table S1 and quoted for C_2v_ symmetry.

Figure 1 shows the spectra of fragment ion pairs compared to a more highly resolved double ionization spectrum acquired earlier^22^. The bands in this spectrum due to the direct population of SO_2_^2+^ states located in the Franck–Condon (F.C.) zone accessed from SO_2_(X^1^A_1_) have been identified in previous work^9^, and again in the present computations. Whatever the equilibrium geometry of the SO_2_^2+^ dication, the electronic states are labelled in the C_2ν_ point group since single-photon ionisation occurs from the bent ground neutral SO_2_ (X ^1^A_1_) state. We also mapped the multi-dimensional potentials of the doubly charged ions in the C_s_ point group (cf. SI for more details). Dissociation following the most abundant ion pair pathway of O^+^ + SO^+^ formation sets in at the very onset of double ionization near 34 eV and persists over most of the range of ionization energies. Of the distinct features of the overall spectrum, this charge-separation pathway follows from population of the prominent vibrationally resolved state at 35.3 eV, identified as 1^1^A_2_^9^, but apparently not from population of the states represented by the bands centered at 36.5 and at 37.5 eV. The onset of the charge retaining pathway forming SO^2+^ coincides with the peak of the resolved band, and also with the thermodynamic threshold for O + SO^2+^ at *ca.* 35.46 eV (Table S3). This channel is populated with substantial quantum yield (about 1/3 of the O^+^ + SO^+^ yield) at all energies above threshold, and specifically by decay from a state or states between 37 and 38 eV, seen as a broad peak in the resolved spectrum and apparently correlated with the population of the 3^1^A_1_ (4^1^A′ in C_s_ symmetry) state. Contributions of the triplet states lying in the same energy range cannot be ruled out, in particular the {1^3^A_1_, 2^3^B_1_, 2^3^B_2_} set of states. Of the channels not shown in Fig. 1, the one leading to the three-body products O^+^ + S^+^ + O (cf. Fig. S2), sets in weakly at ~ 37 eV (thermodynamic threshold at 35.1 eV) and shows no distinctive features. These products are formed by sequential decay of primary SO^+^, as reflected in Newton diagrams^23^. Its onset energy agrees with the limit for ground-state products plus the observed total kinetic energy release (KER). Channel (3) will be discussed in a separate paper.

Insights into the fragmentation mechanisms can be obtained from the kinetic energies released in the fragmentations. The KER magnitudes can be extracted from TOF peak widths and shapes, and for the charge-separating channels this has been done several times before^23,24^ with generally concordant results, but without initial state selection. The most detailed measurements made possible by use of a position–sensitive ion detector^23^, gave the full KER distributions for 40.8 eV photoionization. Although we can accurately select ranges of ionization energy (IE), the present experimental data are not ideally suited to the extraction of KERs. In contrast to previous experiments where a continuous light source could be used^24^, in our set-up there has to be a relatively long delay between the photoionisation event and ion extraction (to ensure that even the slowest electrons leave the source region), ions spread out in the source and the TOF peaks get broadened. Nevertheless, we can estimate the mean KEs released from dications formed in limited ionisation energy ranges. To use the available statistics efficiently we have extracted mass spectra from 1 eV wide IE ranges which correspond quite well to the main features in Fig. 1. Results are given in Table S4.

For the charge separation reaction forming O^+^ + SO^+^ we find that the mean KER varies slightly, between 4.5 ± 0.2 to 4.8 ± 0.2 eV (cf. Table S4) over the range of ionization energies (IEs) from 34 to 40 eV. For this two-body dissociation the distributions are quite narrow (FWHM ca. 2 eV) and centered at about 5 eV. When the kinetic energy release is added to the thermodynamic threshold energy, the result (29.6 + 4.5 eV = 34.1 eV) agrees with the observed onset energy. This proves that the products are formed in their ground states as SO^+^(X^2^Π) + O^+^(^4^S_u_), at least at threshold.

For the reaction(s) producing SO^2+^ the KER is much smaller and produces roughly triangular peak shapes, as is known from the work of Field and Eland^24^ (their Fig. 5), where a full list of SO^2+^ KERs as a function of ionization energy range (Table 2 in Ref.^24^) is also presented. The KER values given in the present work agree satisfactorily with the more complete values given in the past^24^. In the 34–40 eV range, this two-body channel SO^2+^ + O shows two distinct energy distributions: (i) a close to zero KER (0.0–0.2 eV) for 35 < IE < 37 eV range and ~ 0.5 eV for 37 < EI < 40 eV (cf. Table S4). These KERs are derived from the peak widths (FWHM) by first subtracting the thermal width assuming that it and the release width add quadratically. There is a clear increase in energy release as the IE increases, but as the absolute magnitudes are uncertain, we give no error limits. It is apparent from Fig. 1 that production of the SO^2+^ ion starts at the estimated thermodynamic limit for its formation, 35.3 eV. The estimate depends on a theoretical value for the double ionization ionisation energy of SO^25^, but this is sufficiently accurate to confirm that ground-state products, SO^2+^(X^1^Σ^+^) + O(^3^P) must be formed at threshold. The rise in KER as a function of IE seems continuous, with no special excursion at the energy of the state(s) near 37.5 eV, which decay specifically to these products.

**Figure 2:**
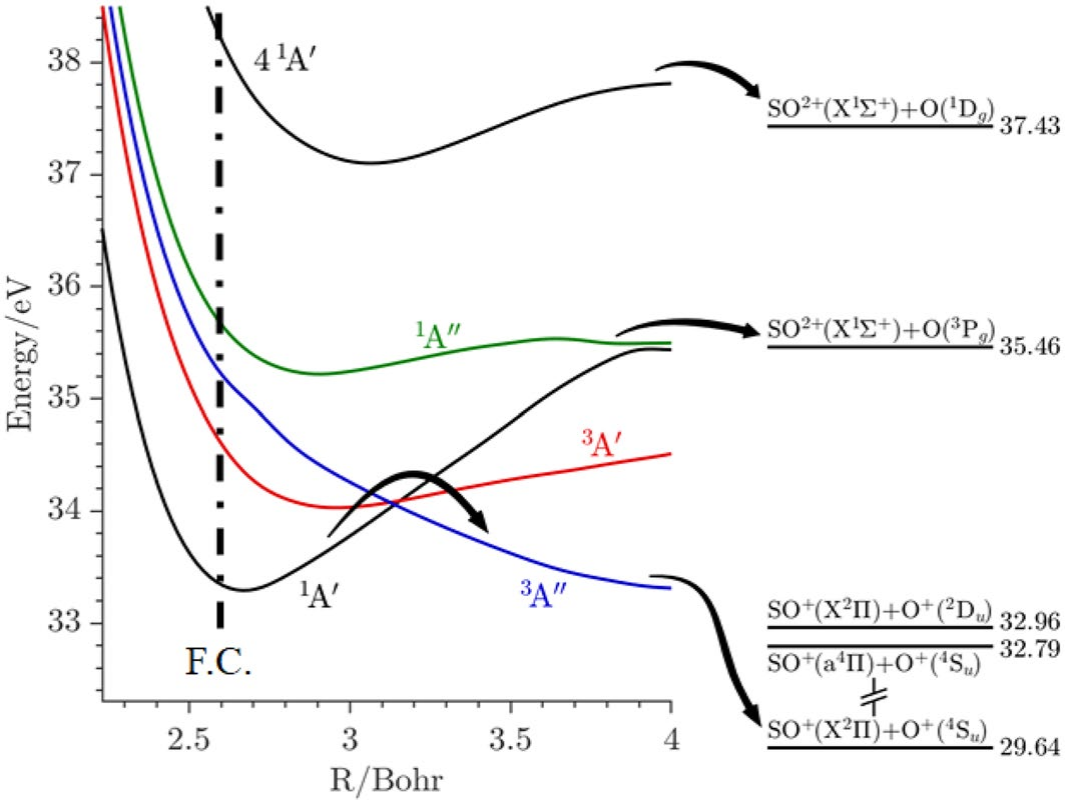
Minimal energy paths (MEPs) along the SO distance (R) for the ^1^A′, ^1^A″, ^3^A′ and ^3^A″ PESs. These MEPs are deduced from the SO_2_^2+^ PESs (Figs. S3–S6) by looking for the minimal energy for each SO distance along the different OSO angles. We show also the 4^1^A′ potential for R_SO_ = 2.7 Bohr and Θ = 120°. The asymptotes are located using the data from Table S3. The dashed dotted vertical line corresponds to the middle of the Franck–Condon (F.C.) region for the SO_2_(X^1^A_1_) → SO_2_^2+^  + 2e^-^ transition. The arrows illustrate the fragmentations discussed in the text.

To investigate the fragmentation dynamics of the SO_2_^2+^ ions more deeply we have performed ab initio computations on the potential energy surfaces (PESs) of the singlet and triplet mainly two-hole (2 h) electronic states located in the whole 33–40 eV range above SO_2_(X^1^A_1_). These computations are done at the CASSCF/MRCI/aug-cc-pV(Q + d)Z level for a wide range of nuclear configurations (cf. Figs. S3–S6), which show a high density of states, consistent with the expected number (20 to 30) of 2 h orbital combinations. Also, we anticipate the existence of a larger number of three-hole-one particle (3h-1p) states accessible in the same regions. Such a high density of electronic states normally implies multiple mutual interactions by vibronic and spin–orbit interactions. In the main dissociation channel, for example, no singlet states correlate to the lowest asymptote, SO^+^(X^2^Π) + O^+^(^4^S_u_), so intersystem crossings (from singlets) and internal conversions (from higher triplets) are needed to populate the lowest triplet that leads to these products (Fig. 2). The formation of the other products, in particular SO^2+^ + O, apparently from specific states is intriguing since the surfaces leading to these products are embedded in a dense manifold of surfaces, mostly leading to the lowest dissociation channel (cf. Table S3 of the SI).

To identify the SO_2_^2+^ electronic states decaying to SO^+^ + O^+^ and to O + SO^2+^ and to understand their dissociation pathways we take inspiration from the earlier calculations^9^, and also make use of the present theoretical data. For the O^+^ + SO^+^ channel, the KERs are between 4.5 and 4.8 eV whatever the initial SO_2_^2+^ state. These experimental values are close to the computed KER where the products are formed in their electronic ground states O^+^(^4^S) + SO^+^(X^2^Π) via the following mechanisms (Fig. 2):

(i) Internal conversions within the singlets manifold of states populating the lowest ^1^A′ state.
(ii) Spin–orbit conversion from this ^1^A′ to the lowest ^3^A″ potential. This triplet can be also reached via internal conversion within the triplets.
(iii) Afterwards, the lowest triplet ^3^A″ leads to the O^+^(^4^S_u_) + SO^+^(X^2^Π) products. The spin–orbit integrals presented in Fig. S7 have magnitudes of several cm^-1^, which are large enough for such internal conversions to be rapid.

The fact that O^+^ + SO^+^ continues to be formed over the whole range of ionisation energies up to 40 eV with little change in kinetic energy release means that the products must be created with increasingly large amounts of internal energy. Only a limited amount of energy can be accommodated as vibration in undissociated SO^+^^26^, so much of the excess energy must be electronic excitation of the product ions. This is consistent with the observation of the sequential formation of O^+^ + S^+^ + O and of optical emission in coincidence with the O^+^ + SO^+^ ion pair with a significant quantum yield^27^, attributed particularly to SO^+^(A^2^Π → X^2^Π). It seems to indicate that internal conversions and intersystem crossings within the SO_2_^2+^ state manifold, although dominant, are not quite complete.

For the SO^2+^ + O channel, we refer to Fig. 2, which presents the minimal energy paths (MEPs) of the ^1^A′, ^1^A″, ^3^A′ and ^3^A″ PESs along the SO distance, and to the KERs listed in Table S4. For IE < 37 eV, computations suggest the production of these fragments occurs on the ^1^A′ and/or the ^1^A″ MEPs. For ^1^A′, we find a Morse-like potential that leads to a plateau at intermediate bond extension (ca. 4 Bohr) very close in energy to the SO^2+^(X^1^Σ^+^) + O(^3^P_g_) asymptote. We also found a ^1^A″ potential with a small potential barrier (of 0.04 eV) before a level at almost exactly the same energy. We suggest that production of ground-state SO^2+^ may occur by intersystem crossing from these surfaces to one of the triplet surfaces correlated to SO^2+^(X^1^Σ^+^) + O(^3^P_g_), which are expected to be relatively flat at these distances in the presence of charge-induced dipole attraction and the absence of Coulomb repulsion. At such distances other competitive curve crossings, which could lead to eventual SO^+^ + O^+^ production are less frequent (cf. Figs. S3–S6). With this interpretation, the computed KERs and the measured ones are the same within the error bars. But in the F.C. region the MEPs concerned are iso-energetic with high vibronic levels of SO_2_^2+^(X^1^Σ_g_^+^/X^1^A_1_) and levels of the vibrationally resolved 1^1^A_2_ state in regions where there are multiple curve crossings. There is also a weak peak at 35.7 eV on the SO^2+^ yield curve in Fig. 1 which suggests that some vertically accessed levels of the resolved 1^1^A_2_ state at 35.3 eV, decay into this channel rather than to the lowest energy products. It follows that to support the proposed mechanisms, we still need an explanation of how the long O–SO bond extension required can be reached in significant yield, without undergoing prior conversion to lower energy states.

For production of SO^2+^ in the range of IE above 37 eV, we measure a KER of ~ 0.5 eV. This value is sufficiently different from the previous case to suggest that a different potential surface is involved. Figure 2 shows that the formation of this product coincides with the occurrence of the SO^2+^(X^1^Σ^+^) + O(^1^D_g_) asymptote where there is no difficulty over the spin. Computations indicate that the SO_2_^2+^(4^1^A′/3^1^A_1_) state which leads directly to these products can be populated by direct ionization in the F.C. zone. Figure 2 shows that the SO_2_^2+^(4^1^A′/3^1^A_1_) surface exhibits a potential barrier toward dissociation of ~ 0.4 eV, which is in good agreement with the measured KER at these IEs (cf. Table S4). Again, for this route to be followed in the high observed yield after direct vertical double ionisation in the F.C. zone, the expected competitive internal conversions would need to be somehow turned-off to allow the necessary bond extension. Because this seems both ad hoc and inherently unlikely, we looked for another explanation.

The clue to an alternative mechanism is given by the observation in double ionisation of autoionising O* over the range of ionisation energy from near threshold right up to the photon energy, as visible in the electron pair coincidence map^9^ and shown in more detail in Fig. S8. The observation of O* autoionization over the whole energy range proves that super-excited states of SO_2_^+^* are also populated over the same wide energy range to act as precursors. There is indeed no lack of suitable states, because below each of the 20 to 30 2-h states of SO_2_^2+^ (Figs. S3–S6) there is a quasi-infinity of singly-charged 2h-1p Rydberg states in series converging on those dication states. The high Rydbergs have long lifetimes (proportional roughly to n*^3^) towards both optical emission and autoionisation^28^. The cores of the higher Rydberg states, being doubly ionised, excited and hardly influenced by the attached electron, will start to dissociate in the same way as bare SO_2_^2+^ ions. Many will follow the most favorable path of O^+^–SO^+^ bond extension leading to breakage. A departing O^+^ may then capture the Rydberg electron on its way out, producing the autoionisating O* states observed. The same or other Rydbergs, some with shorter but still relatively long lifetimes to autoionisation, will undergo significant O–SO bond extension before production of nascent SO_2_^2+^. The new geometry at the moment of autoionisation can bring the molecular dications into regions of parameter space where curve-crossings and the consequent state conversions are rare, allowing the formation of energetically disfavored products with apparent state selectivity, exactly as is seen in the case of SO^2+^. At the same time, the molecular autoionisation will contribute to the whole double ionisation spectrum, adding to the direct vertical process. This mechanism should be operative in all molecular double ionisation by photon or electron impact not just in SO_2_.

The present technique is the only existing means of acquiring such detailed information on dicationic decays from the states of ions formed by photoionisation. We note that similar information for the different set of mainly singlet states accessed by the Auger effect could be acquired by Auger-electron–ion coincidence techniques. There are only six other molecules whose fragmentation after double ionisation can be compared with that of SO_2_. In BrCN^2+^, ICN^2+^^29^ and CF_3_I^2+^^30^ decays no state-specific behavior can be seen. The N_2_O^2+^ ion decays radiatively from specific states, but it shows no other state-specific decay behavior. The CS_2_^2+^ ion both emits fluorescence from two specific excited states and also decays to C^+^ + S_2_^+^ from states in a limited energy range^31^, and is the closest to SO_2_^2+^ in respect of this behavior. The electron–electron coincidence map for CS_2_ at 40.81 eV shows evidence of autoionisation^29^ akin to that in SO_2_, further confirming the validity of the proposed mechanism.

We have here identified a new non-conventional mechanism involved in the unimolecular decomposition of doubly charged molecular dications which accounts for the observation of energy-disfavored dissociation channels with substantial quantum yield in the decay of doubly ionized SO_2_. The mechanism almost certainly operates more widely. In the case of SO_2_, we have identified particular electronic states as probable surfaces upon which the dissociations to high energy products occur. The mechanism is one of indirect ionisation, akin to the usual Auger effect, providing a non-vertical overall pathway to double ionisation of molecules, outside the F.C. zone. In many cases the outcome will be indistinguishable from vertical double ionization in the Franck–Condon region, but in some cases it can permit energetically disfavored pathways to be followed to a much greater extent than would be expected on the basis of free internal energy flow.

## Experimental method

Multi-electron multi-ion coincidence experiments were done at 304 Å (40.8 eV), well above the estimated adiabatic single-photon double ionization onset 33.5 eV^9^. The TOF-PEPEPIPICO apparatus which has been described before^27^ allows continuous (simultaneous) detection of two electrons with energy measurement and two positive ions with mass/charge measurement. In brief, ionisation occurs where molecules in an effusive jet from a hollow needle intersect wavelength-selected light from a pulsed discharge in He. At the moment of ionisation there is a weak electric field in the source region which accelerates any near-zero energy photoelectrons towards a 2 m distant detector, giving them sufficient velocity to arrive there in less than 10 μs. A continuous, divergent strong magnetic field in the same region guides almost all photoelectrons, irrespective of kinetic energies of less than a few hundred eV, into a solenoidal field which carries them all to the detector. After about 150 ns, when all relevant electrons have left the source region, a strong electric field pulse extracts ions in the opposite direction through a two-field time-of-flight mass spectrometer to a separate detector. The strength of the ion drawout pulse and the associated acceleration field, as set for time-focus conditions, determine the mass resolution, and the time-width of signals for fragment ions formed with more than thermal kinetic energies. The drawout pulse field strength affects the detectability of ion pairs of equal mass-to-charge ratio (m/z) such as O^+^ + O^+^ or O_2_^+^ + S^+^ because the ion detector and associated electronics used in these experiments imposed a deadtime of 50 ns.

At the time of these experiments the efficiency was about 30% for electrons and 10% for ions at m/z = 100. Because the final ion energy at the detector was less than 2 keV, the efficiency was somewhat higher for lighter ions. Electron resolution (E/ΔE) was about 20 throughout, while mass resolution ranged from 30 to 100 (FWHM) according to the strength of the drawout pulse; all ions of interest from SO_2_ except equal mass pairs were fully resolved under all conditions, but the ^34^S isotopic variants were only partially resolved.

## Theoretical methods

All the ab-initio electronic calculations with different levels of theory were performed using the MOLPRO program suite^32^. In the present study, we mapped the SO_2_^2+^ six lowest A′ and six lowest A′′ three-dimensional potential energy surfaces (PESs) of singlet and triplet spin multiplicities along the SO distance and the in-plane angle, θ = OSO varying from 40° to 180°. The other SO distance is kept fixed at 2.7 Bohr i.e. its value in SO_2_(X^1^A_1_) at equilibrium.

The PESs have been generated using the Complete Active Space Self Consistent Field (CASSCF)^33,34^ approach followed by the internally contracted multi reference configuration interaction method (MRCI)^35–37^. The CASSCF active space is constituted by the whole set of all configurations which is allowing all the possible excitations of all valence electrons in valence orbitals. The electronic states having the same spin multiplicity are computed according to the state-average procedure, as implemented in MOLPRO. For MRCI calculations, all configurations having a coefficient greater than 0.005 in the CI expansion of the CASSCF wave functions were taken into account as a reference. The computations were done in the C_s_ point group with wave functions leading to more than 7 × 10^8^ uncontracted configurations for the singlet electronic states. For the triplet electronic states, we considered more than 26 × 10^8^ uncontracted configurations. For these calculations, the aug-cc-pV(Q + d)Z and the aug-cc-pVQZ basis sets of Dunning et al. were used to describe the S and O atoms, respectively^38–40^. In fact, adding the tight-d functions to describe the sulfur atom improve the accuracy of the results as shown in different works^41–43^. Furthermore, to investigate the spin-obit coupling at the crossings between some electronic SO_2_^2+^ states, the spin orbit integrals where computed using the CASSCF wave functions and the uncontracted spd cc-pVTZ basis set. We used the Breit–Pauli spin–orbit Hamiltonian as implemented in MOLPRO. Indeed, the spin–orbit integrals were evaluated, in Cartesian coordinates, over the CASSCF wavefunctions, where the effective Breit–Pauli SO operator, H_SO_, as implemented in MOLPRO was used^44^.

To evaluate the energies of the dissociation limits of SO_2_^2+^ forming the [SO + O]^2+^, the partially spin restricted coupled cluster method including perturbative treatment of triple excitation (RCCSD(T))^45–48^ was used. For the RCCSD(T) computations we used the aug-cc-pV(5 + d)Z basis set for sulfur and the aug-cc-pV5Z for oxygen.

## Supplementary Information

Supplementary Information.

## Data Availability

The experimental and theoretical datasets generated during and/or analysed during the current study are available from the corresponding authors on reasonable request.
