# Supplementary material for: State selective fragmentation of doubly ionized sulphur dioxide
Source: Sci Rep. 2021 Aug 24;11:17137. doi: 10.1038/s41598-021-96405-5 (PMC8384974; doi:10.1038/s41598-021-96405-5)
Supplement: Supplementary file 1 — Supplementary Information. [file 41598_2021_96405_MOESM1_ESM.docx]

**Supplementary material**

State selective fragmentation of doubly ionized sulphur dioxide

M. Jarraya^a,b^, M. Wallner^c^, G. Nyman^d^, S. Ben Yaghlane^b^, M. Hochlaf^a^*, J. H.D. Eland^e^, and R. Feifel^c^*

^a^ Université Gustave Eiffel, COSYS/LISIS, 5 Bd Descartes 77454, Champs sur Marne, France.

^b^ Université de Tunis El Manar, Faculté des Sciences de Tunis, Laboratoire de Spectroscopie Atomique, Moléculaire et Applications – LSAMA, 2092, Tunis, Tunisia.

^c^ University of Gothenburg, Department of Physics, 412 58 Gothenburg, Sweden.

^d^ University of Gothenburg, Department of Chemistry and Molecular Biology, 405 30 Gothenburg, Sweden.

^e^ Oxford University, Department of Chemistry, Physical and Theoretical Chemistry Laboratory, South Parks Road, Oxford OX1 3QZ, United Kingdom

* Corresponding authors: [majdi.hochlaf@univ-eiffel.fr](mailto:majdi.hochlaf@univ-eiffel.fr); [raimund.feifel@physics.gu.se](mailto:raimund.feifel@physics.gu.se).


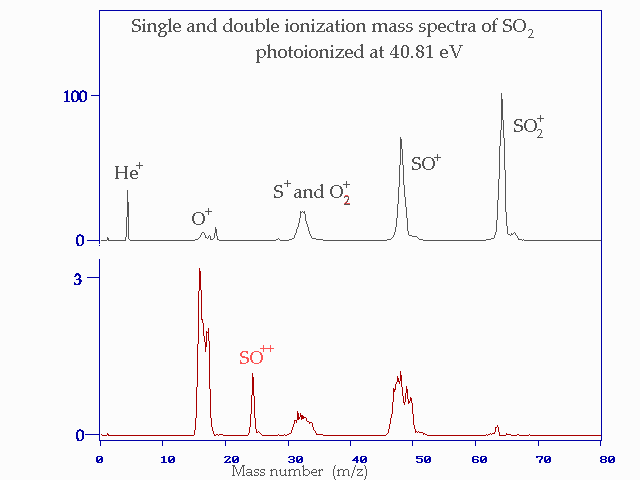


**Figure S1:** Complete single ionization and double ionization mass spectra of SO_2_ from 40.8 eV photoionization. In addition to He (from the gas-discharge light source) a small H_2_O^+^ signal can also be seen in single ionization. The relative intensities in these spectra are strongly indicative but not exact, because of the expected mass-dependent detection efficiency and the suppression of ions of equal mass number by the detection deadtime. The peaks for O^+^ and SO^+^ both show partial splitting into doublets because of time separation of initial forward- and backward–flying ions. If any of SO_2_^2+^, S^2+^ or O^2+^ were present in any quantity they would appear as sharp spikes on the baseline or on top of the broad peaks for singly-charged species.

**Figure S2:** Fourfold coincidence spectra showing counts of electron pairs with the indicated ion pairs after photoionization of SO_2_ at 40.81 eV photon energy.

**Table S1:** MRCI/aug-cc-pV(Q+d)Z vertical double ionisation energies of SO_2_^2+^ quoted with respect to the energy at the equilibrium geometry of SO_2_(X1A1) (i.e. OSO angle = 120° and SO distances = 2.7 Bohrs).

| Electronic state | T (eV) |
| --- | --- |
| 1^1^A_1_ | 34.25 |
| 1^3^B_2_ | 34.33 |
| 1^3^A_2_ | 34.90 |
| 1^1^A_2_ | 35.40 |
| 1^1^B_2_ | 35.46 |
| 1^3^B_1_ | 36.01 |
| 1^1^B_1_ | 36.49 |
| 2^1^A_1_ | 36.69 |
| 1^3^A_1_ | 37.62 |
| 3^1^A_1_ | 37.70 |
| 2^3^B_1_ | 37.73 |
| 2^3^B_2_ | 37.78 |
| 2^1^A_2_ | 38.25 |
| 2^3^A_2_ | 38.41 |
| 3^3^B_2_ | 38.49 |
| 2^1^B_1_ | 38.53 |
| 4^3^B_2_ | 38.80 |
| 2^3^A_1_ | 39.08 |
| 4^1^A_1_ | 39.23 |
| 2^1^B_2_ | 39.34 |
| 3^3^A_2_ | 39.43 |
| 3^3^B_1_ | 39.52 |
| 3^1^A_2_ | 39.62 |

**Table S2:** Total energies and structural parameters of the species involved in this work.

| Species | Base | R_1_ / Å | R_2_ / Å | θ / ° | ZPE / eV | E / Hartree |
| --- | --- | --- | --- | --- | --- | --- |
| O^+^(^4^S_u_) | aug-cc-pV(Q+d)Z |  |  |  |  | -74.4982 |
|  | aug-cc-pV(5+d)Z |  |  |  |  | -74.5022 |
|  | CBS |  |  |  |  | -74.5065 |
| O(^3^P_g_) | aug-cc-pV(Q+d)Z |  |  |  |  | -74.9949 |
|  | aug-cc-pV(5+d)Z |  |  |  |  | -75.0004 |
|  | CBS |  |  |  |  | -75.0062 |
| SO_2_(X^1^A_1_) | aug-cc-pV(Q+d)Z | 1.4366 | 1.4366 | 119.27 | 0.19007 | -548.0631 |
|  | aug-cc-pV(5+d)Z | 1.4339 | 1.4339 | 119.31 | 0.19103 | -548.0824 |
|  | CBS | 1.4312 | 1.4312 | 119.36 |  | -548.1027 |
| SO^2+^(X^1^Σ^+^) | aug-cc-pV(Q+d)Z | 1.3899 |  |  | 0.088562 | -471.7691 |
|  | aug-cc-pV(5+d)Z | 1.3875 |  |  | 0.089092 | -471.7791 |
|  | CBS | 1.3849 |  |  |  | -471.7896 |
| SO^+^(X^2^Π) | aug-cc-pV(Q+d)Z | 1.4291 |  |  | 0.081889 | -472.4812 |
|  | aug-cc-pV(5+d)Z | 1.4266 |  |  | 0.082283 | -472.4919 |
|  | CBS | 1.4239 |  |  |  | -472.503 |

**Table S3:** Dissociation energies (E, eV) of SO_2_^2+^ leading to [SO+O]^2+^ fragments laying in the 29 - 40 eV with respect to SO_2_(X^1^A_1_). For SO^+^ and SO^2+^, we used the excitation energies from Refs.^[[1]](#endnote-1),^^[[2]](#endnote-2)^. We also used the data in Table S2 and from Ref.^[[3]](#endnote-3)^.

| Dissociation channel | E |
| --- | --- |
| SO^+^ (X^2^Π) + O^+^ (^4^S_u_) | 29.64 |
| SO^+^ (a^4^Π) + O^+^ (^4^S_u_) | 32.77 |
| SO^+^ (X^2^Π) + O^+^ (^2^D_u_) | 32.96 |
| SO^+^ (A^2^Π) + O^+^ (^4^S_u_) | 33.45 |
| SO^+^ (1^2^ɸ) + O^+^ (^4^S_u_) | 34.34 |
| SO^+^ (b^4^Σ^-^) + O^+^ (^4^S_u_) | 34.36 |
| SO^+^ (X^2^Π) + O^+^ (^2^P_u_) | 34.66 |
| SO^+^ (C^2^Π) + O^+^ (^4^S_u_) | 35.29 |
| SO^2+^ (X^1^Σ^+^) + O (^3^P_g_) | 35.46 |
| SO^+^ (2^4^Π) + O^+^ (^4^S_u_) | 35.59 |
| SO^+^ (1^2^Δ) + O^+^ (^4^S_u_) | 35.64 |
| SO^+^ (B^2^Σ^-^) + O^+^ (^4^S_u_) | 35.81 |
| SO^+^ (a^4^Π) + O^+^ (^2^D_u_) | 36.10 |
| SO^+^ (1^4^Δ) + O^+^ (^4^S_u_) | 36.21 |
| SO^+^ (2^2^Σ^+^) + O^+^ (^4^S_u_) | 36.24 |
| SO^+^ (4^2^Π) + O^+^ (^4^S_u_) | 36.59 |
| SO^+^ (A^2^Π) + O^+^ (^2^D_u_) | 36.77 |
| SO^2+^ (X^1^Σ^+^) + O (^1^D_g_) | 37.43 |
| SO^+^ (1^2^ɸ) + O^+^ (^2^D_u_) | 37.66 |
| SO^+^ (b^4^Σ^-^) + O^+^ (^2^D_u_) | 37.69 |
| SO^+^ (a^4^Π) + O^+^ (^2^P_u_) | 37.79 |
| SO^+^ (A^2^Π) + O^+^ (^2^P_u_) | 38.47 |
| SO^+^ (C^2^Π) + O^+^ (^2^D_u_) | 38.61 |
| SO^2+^ (1^3^Σ^+^) + O (^3^P_g_) | 38.91 |
| SO^+^ (2^4^Π) + O^+^ (^2^D_u_) | 38.92 |
| SO^+^ (1^2^Δ) + O^+^ (^2^D_u_) | 38.96 |
| SO^+^ (B^2^Σ^-^) + O^+^ (^2^D_u_) | 39.14 |
| SO^+^ (1^2^ɸ) + O^+^ (^2^P_u_) | 39.35 |
| SO^+^ (b^4^Σ^-^) + O^+^ (^2^P_u_) | 39.41 |
| SO^+^ (1^4^Δ) + O^+^ (^2^D_u_) | 39.53 |
| SO^+^ (2^2^Σ^+^) + O^+^ (^2^D_u_) | 39.56 |
| SO^2+^ (X^1^Σ^+^) + O (^1^S_g_) | 39.65 |
| SO^+^ (4^2^Π) + O^+^ (^2^D_u_) | 39.91 |
| SO^2+^ (1^3^Δ) + O (^3^P_g_) | 40.06 |
| SO^+^ (C^2^Π) + O^+^ (^2^P_u_) | 40.30 |

**Table S4:** CM kinetic energy releases (KER, eV) for the formation of O^+^ + SO^+^ and SO^2+^ + O products for different ionization energy (IE, eV) ranges.

| Exp. | | |
| --- | --- | --- |
| IE | KER | |
|  | O^+^+SO^+^ | SO^2+^+O |
| 34-35 | 4.5±0.2 | - |
| 35-36 | 4.7±0.2 | 0.02 |
| 36-37 | 4.7±0.2 | 0.24 |
| 37-38 | 4.7±0.2 | 0.51 |
| 38-40 | 4.8±0.2 | 0.55 |
| Theo. | | |
| Dissociation channel | KER | |
| SO^+^(X^2^Π)+O^+^(^4^S_u_) | 5.80 ^a)^; 4.41 ^b)^ | |
| SO^2+^(X^1^Σ^+^)+O(^3^P_g_) | 0.00 ^a)^; 0.04 ^c)^ | |
| SO^2+^(X^1^Σ^+^)+O(^1^D_g_) | 0.36 ^d)^ | |

^a^ On the ^1^A′ minimal energy path. Cf. Fig. 2. ^b^ From the crossing between the lowest ^1^A′ and ^3^A′/^3^A″ states. Cf. Fig. 2. ^c^ On the ^1^A″ minimal energy path. Cf. Fig. 2. ^d^ On the 4^1^A′ (i.e. 3^1^A_1_) state potential. Cf. Fig. 2.


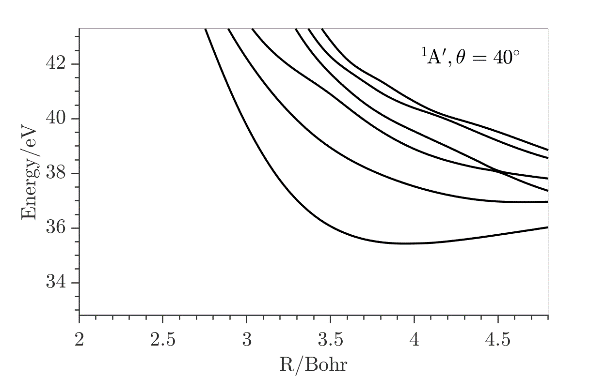

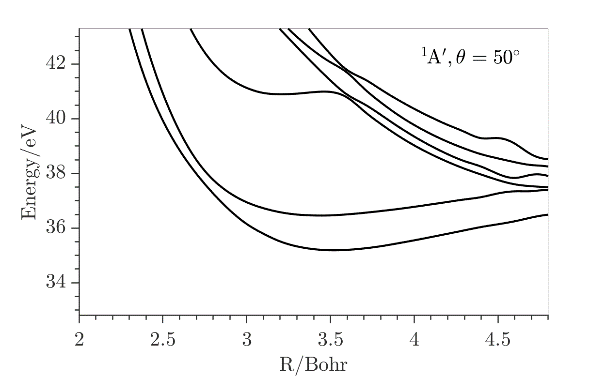

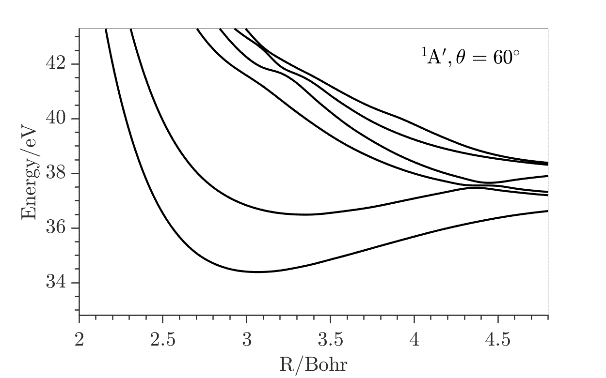

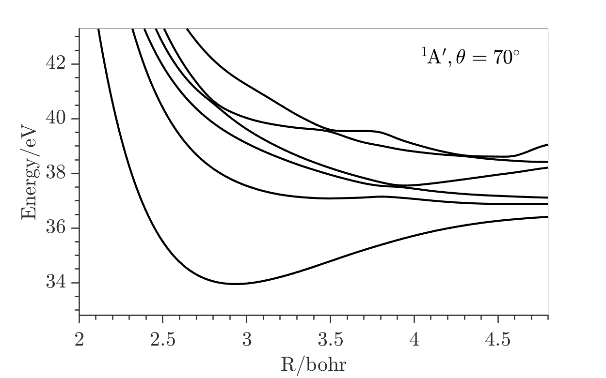

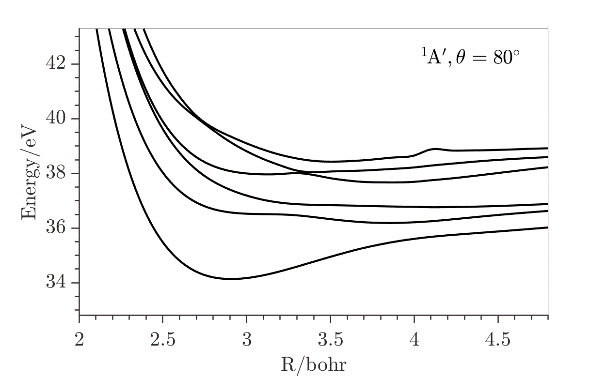

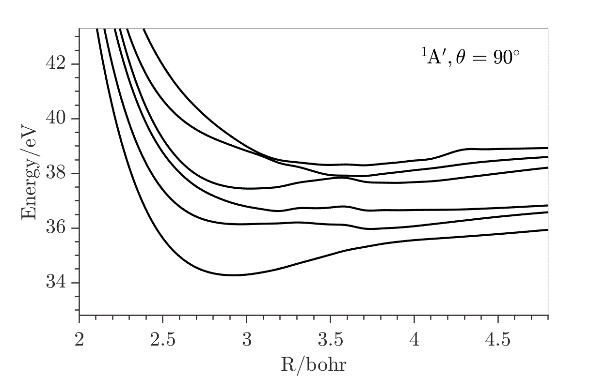

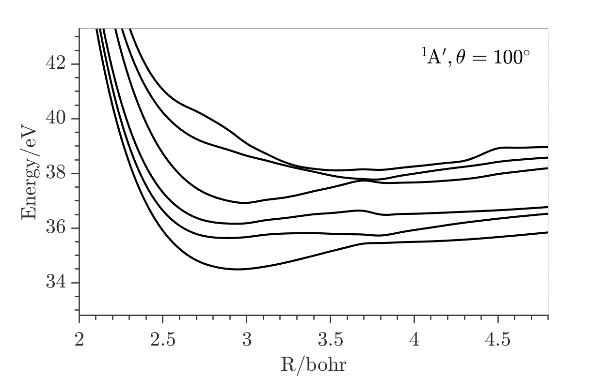

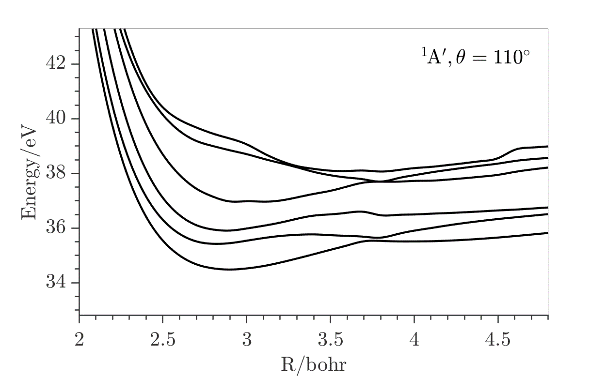

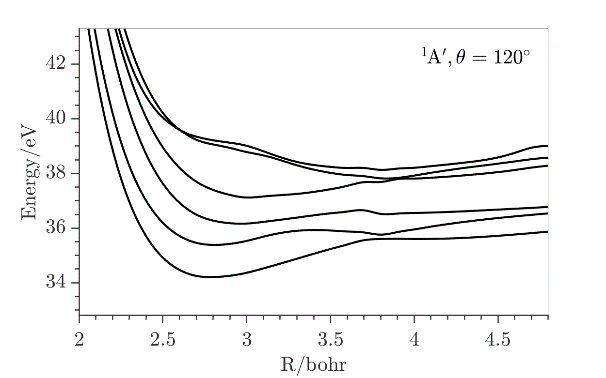

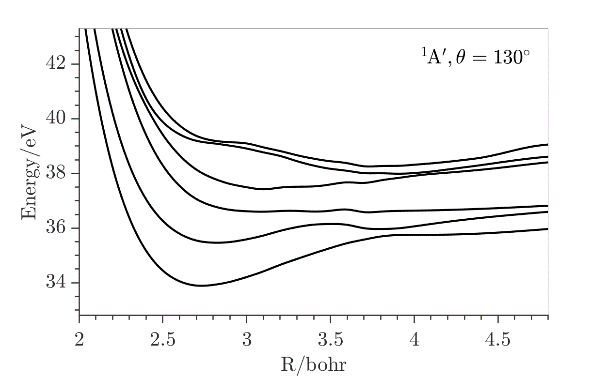

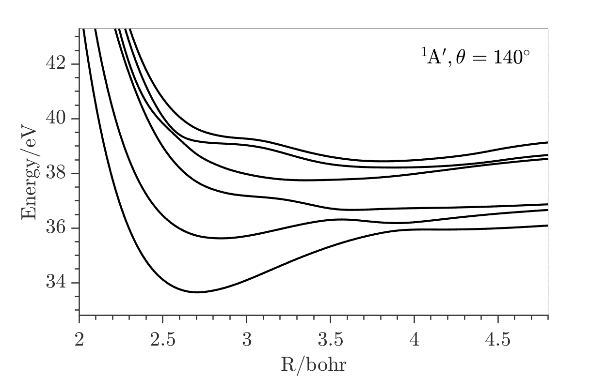

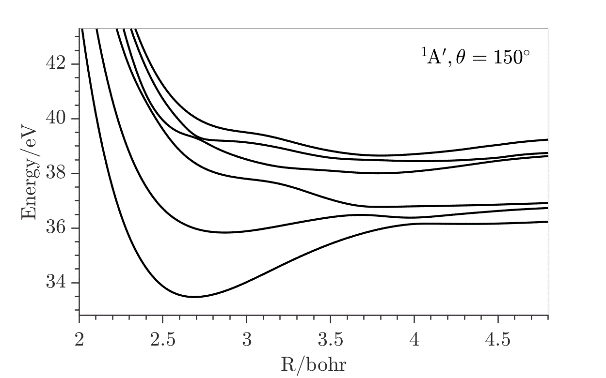

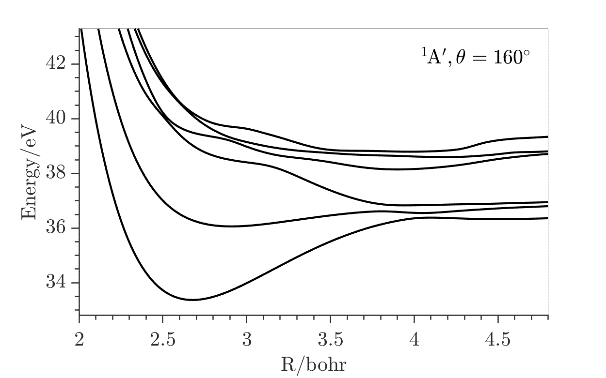

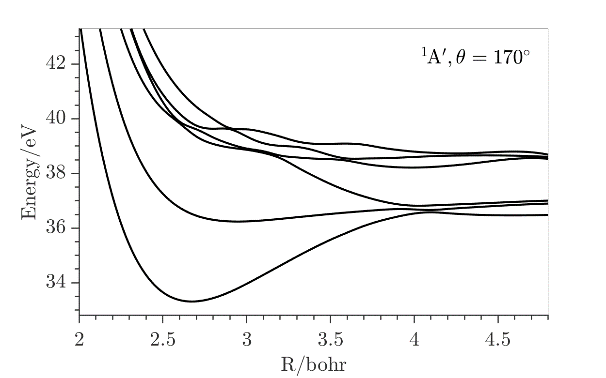

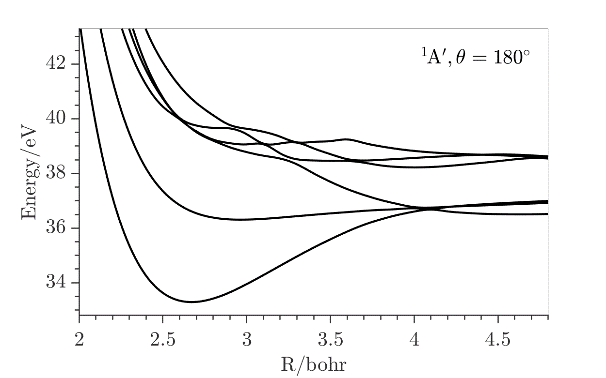


**Figure S3:** One dimensional cuts of the lowest ^1^A′ states of SO_2_^2+^ for in-plane angle θ from 40° to 180° along the SO distance where the other SO is kept fixed at its value in SO_2_(X^1^A_1_) at equilibrium (i.e. 2.7 Bohr). The reference energy is that of SO_2_(X^1^A_1_) at equilibrium.


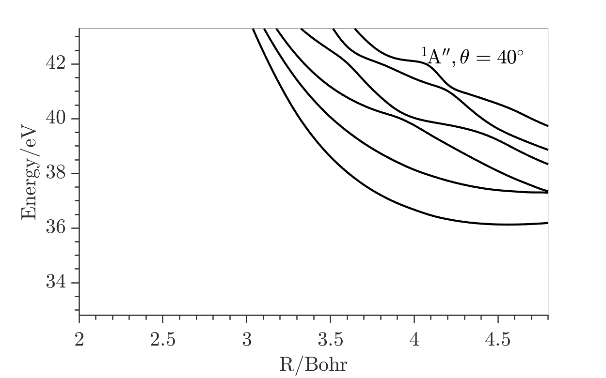

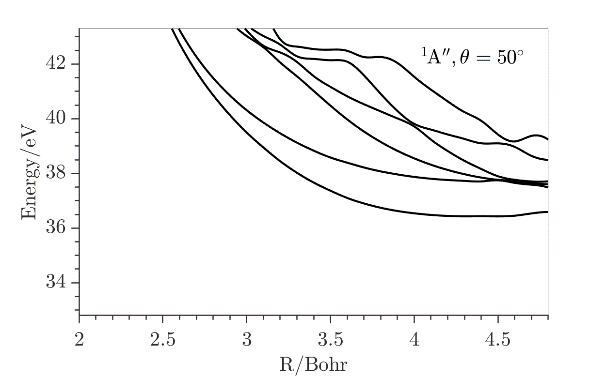

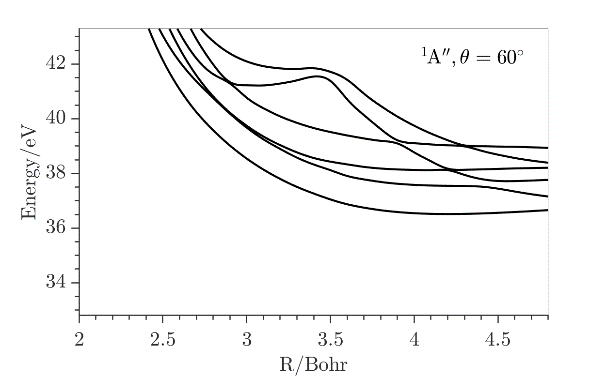

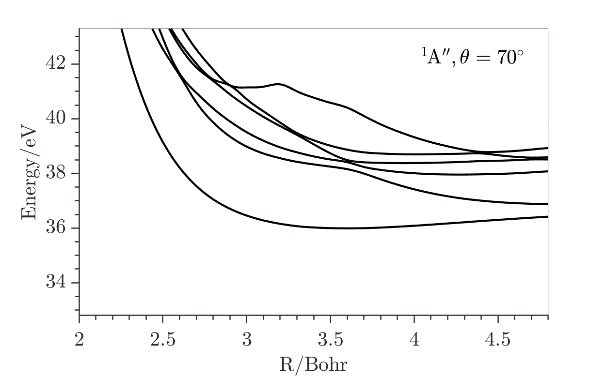

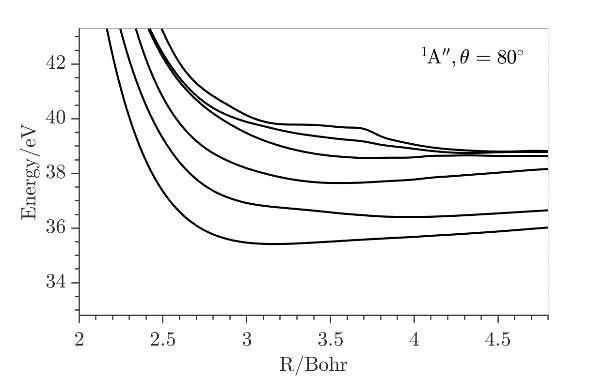

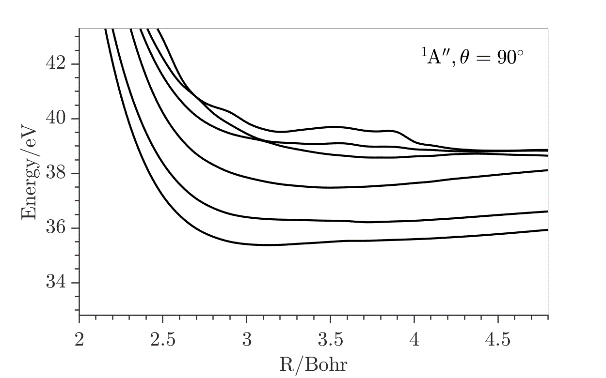

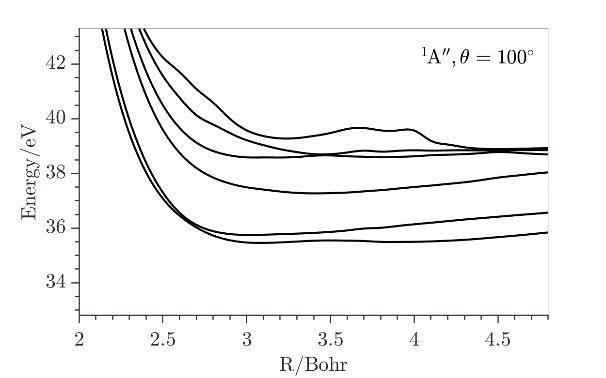

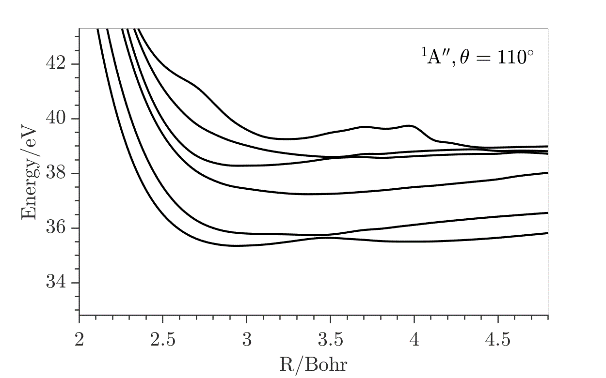

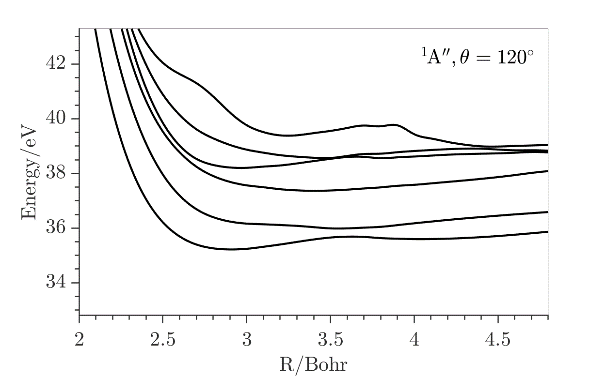

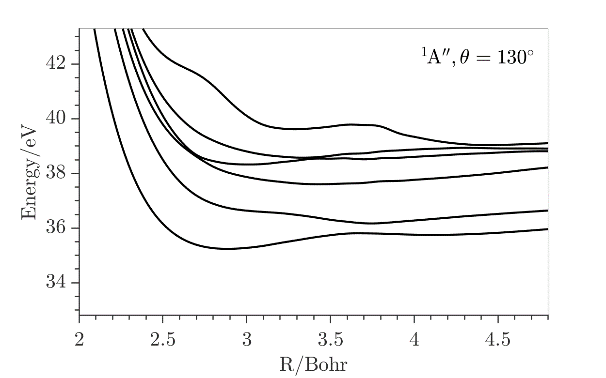

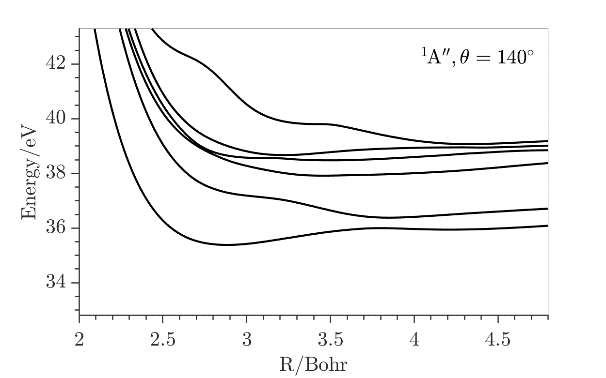

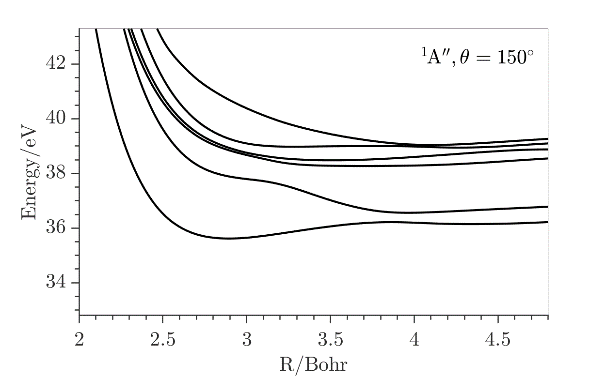

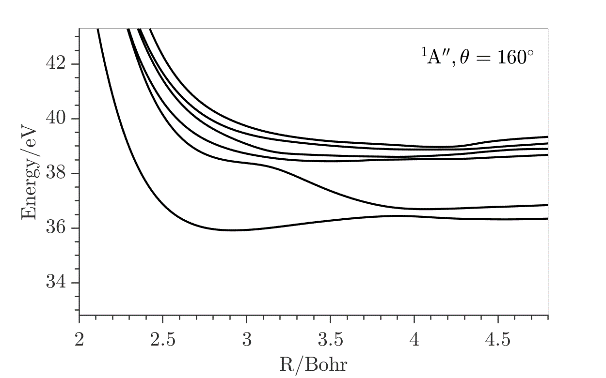

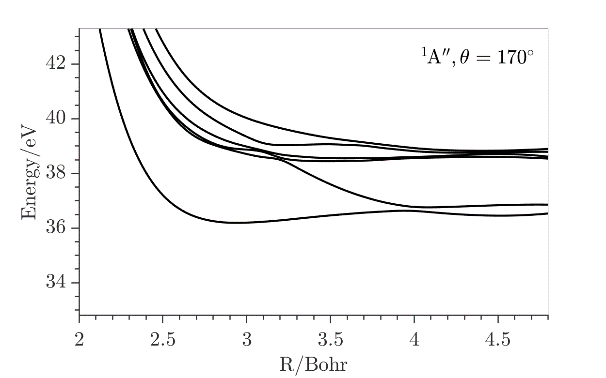

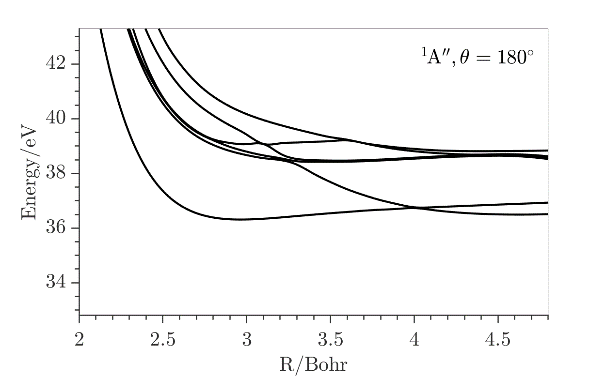


**Figure S4:** One dimensional cuts of the lowest ^1^A′′ states of SO_2_^2+^ for in-plane angle θ from 40° to 180° along the SO distance where the other SO is kept fixed at its value in SO_2_(X^1^A_1_) at equilibrium (i.e. 2.7 Bohr). The reference energy is that of SO_2_(X^1^A_1_) at equilibrium.


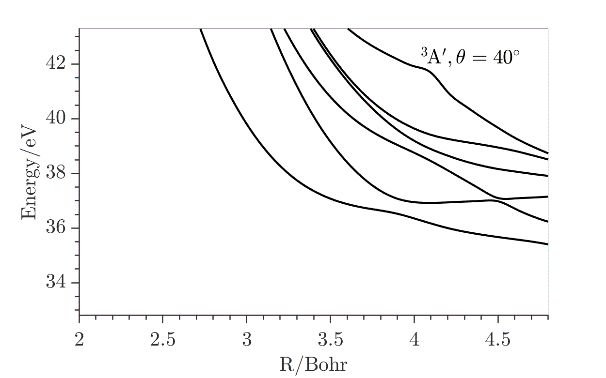

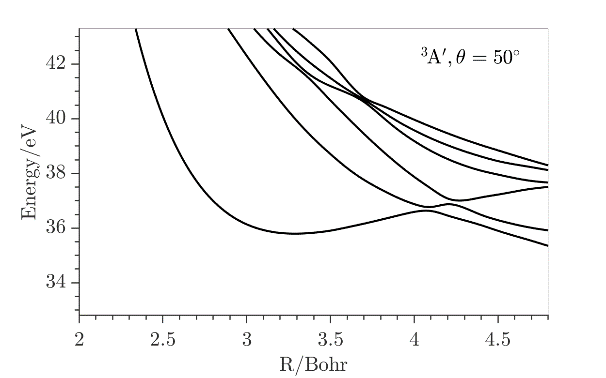

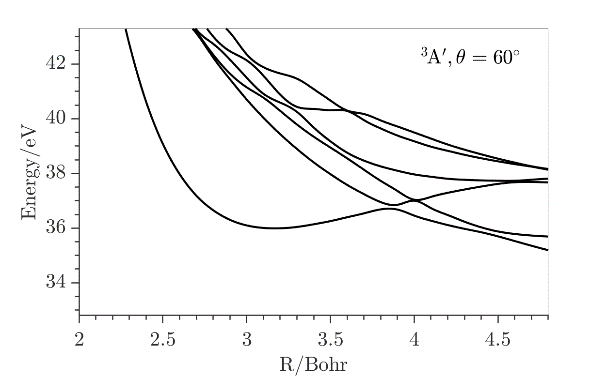

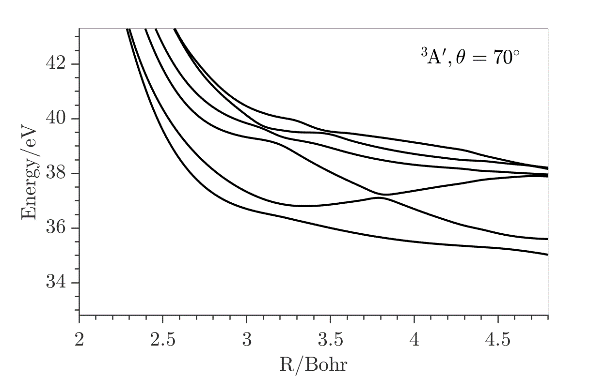

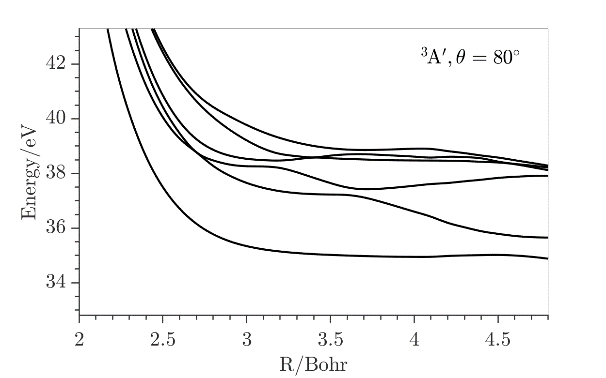

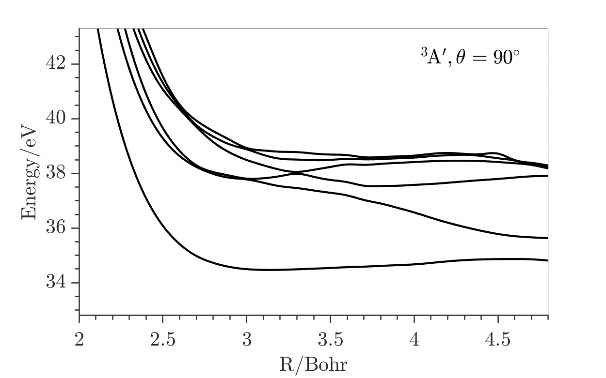

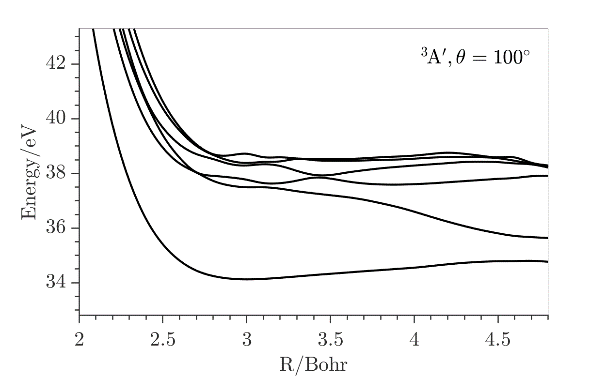

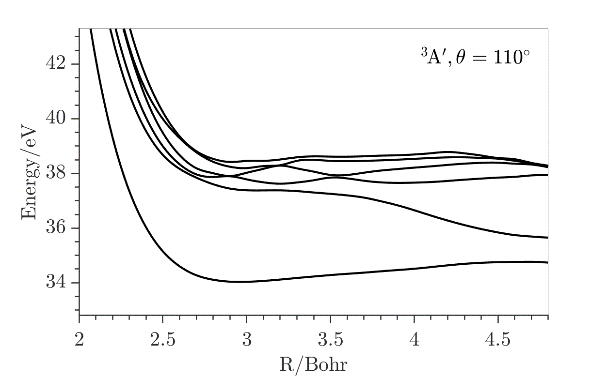

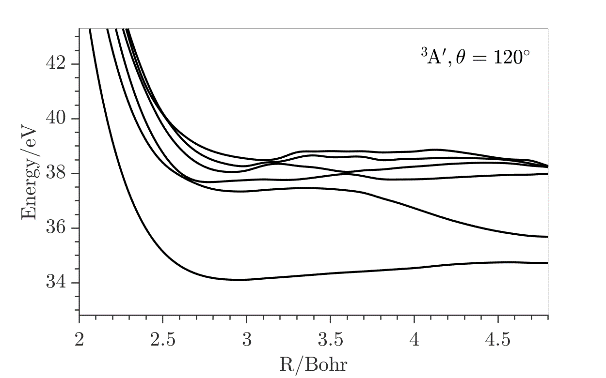

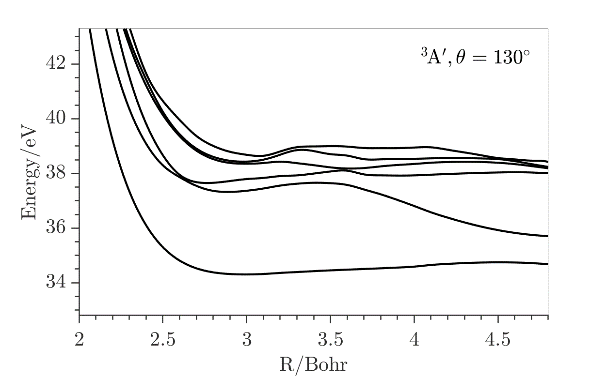

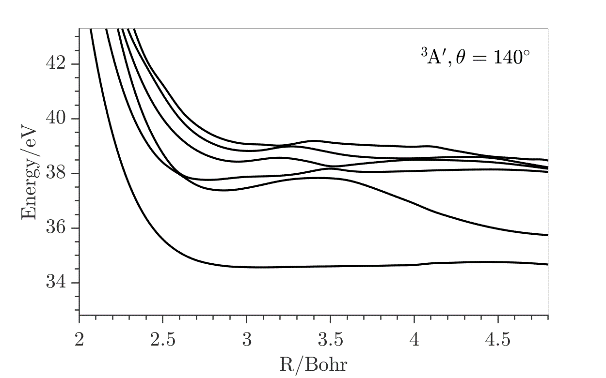

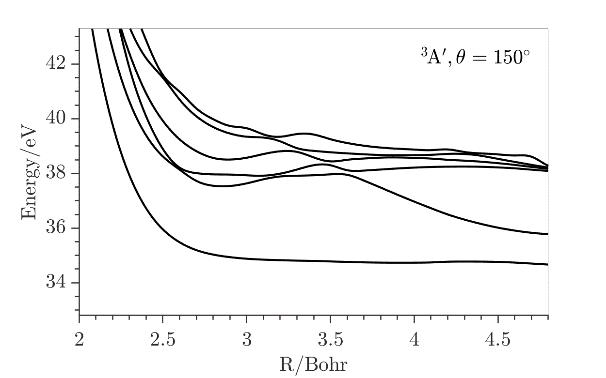

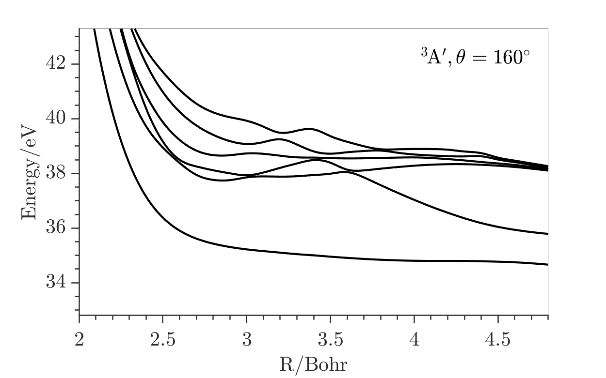

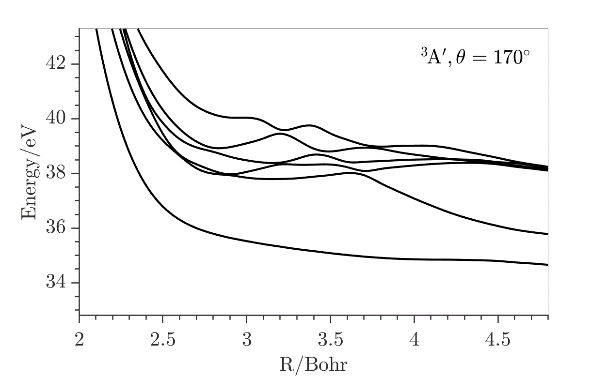

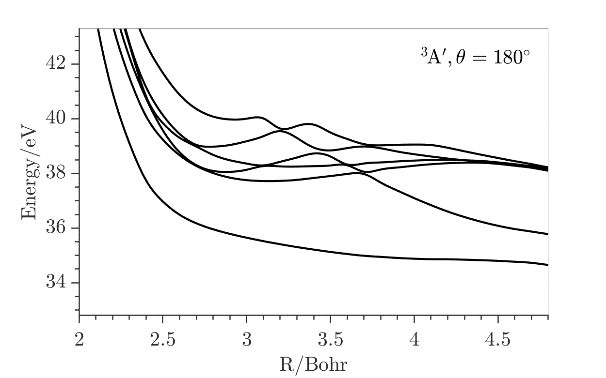


**Figure S5:** One dimensional cuts of the lowest ^3^A′ states of SO_2_^++^ for in-plane angle θ from 40° to 180° along the SO distance where the other is kept fixed at its value in SO_2_(X^1^A_1_) at equilibrium (i.e. 2.7 Bohr). The reference energy is that of SO_2_(X^1^A_1_) at equilibrium.


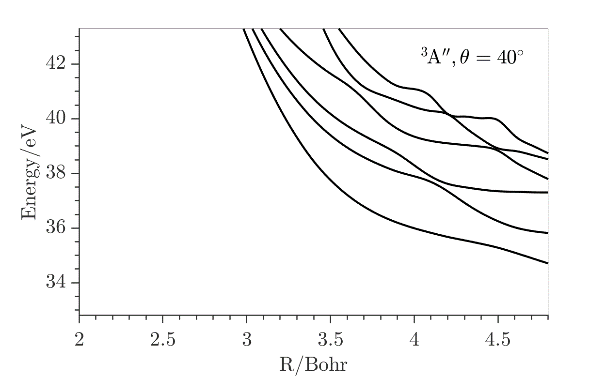

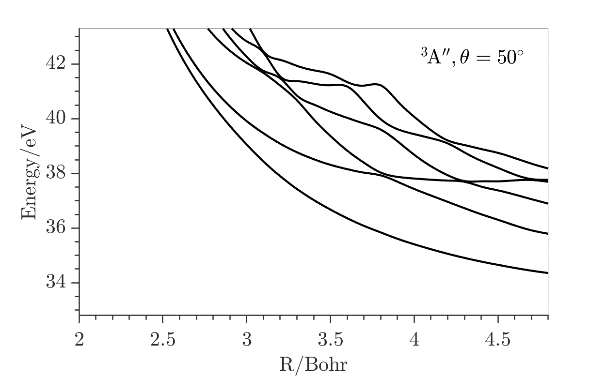

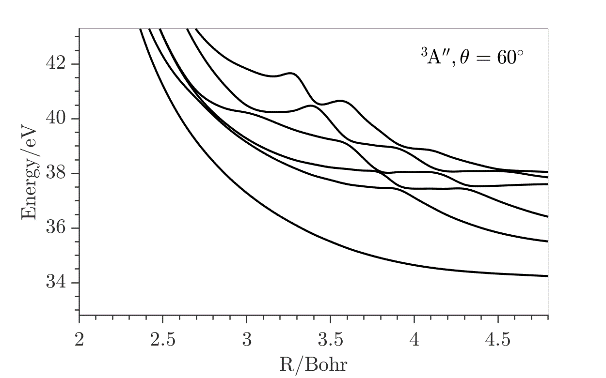

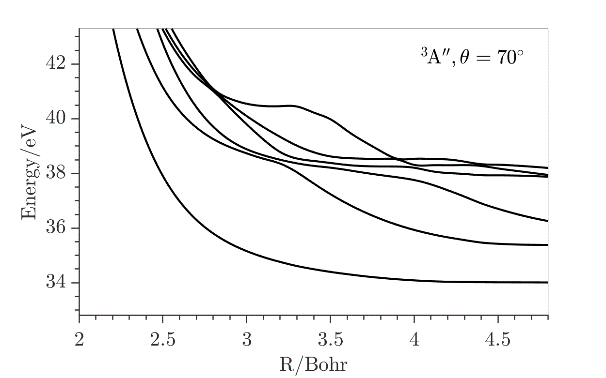

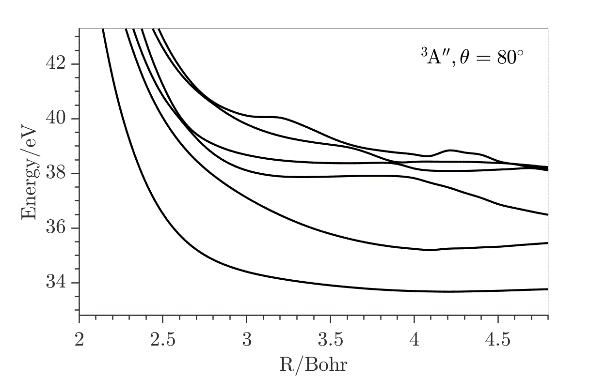

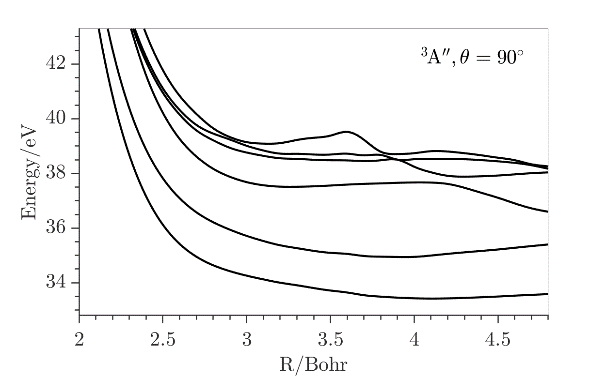

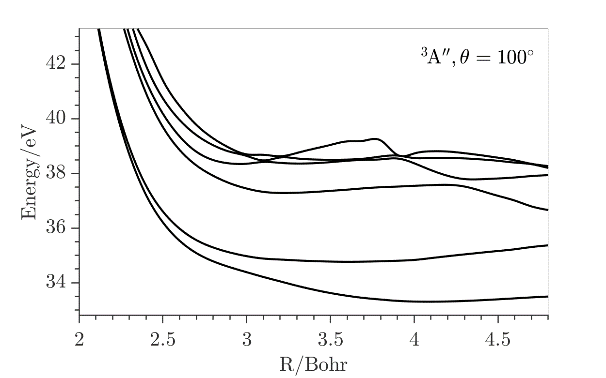

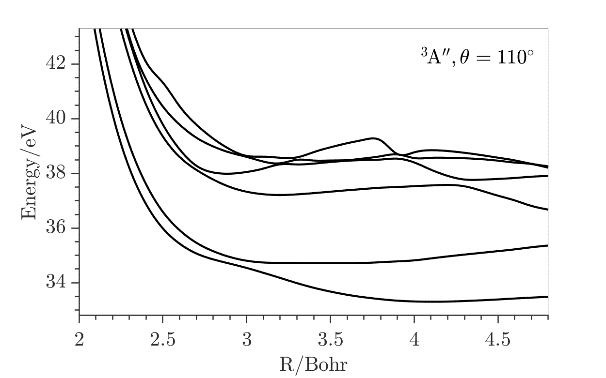

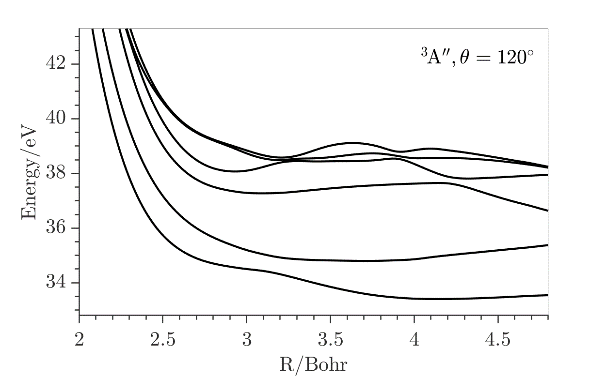

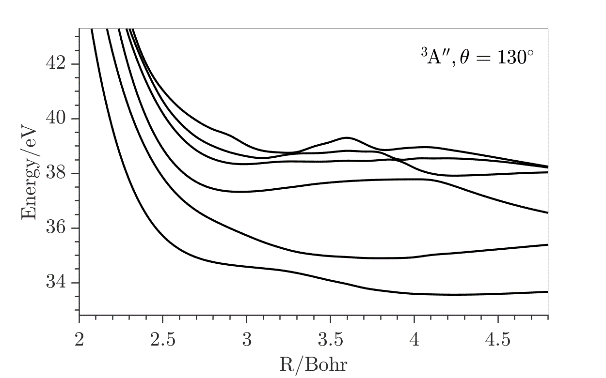

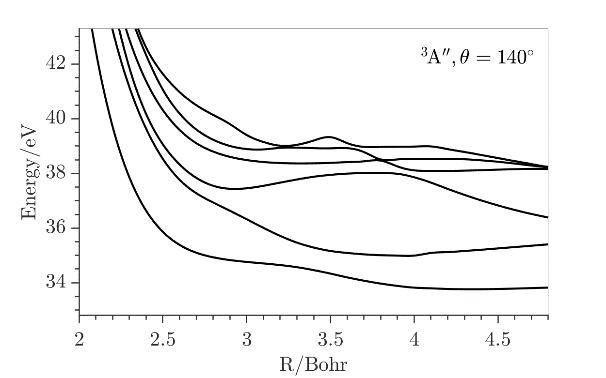

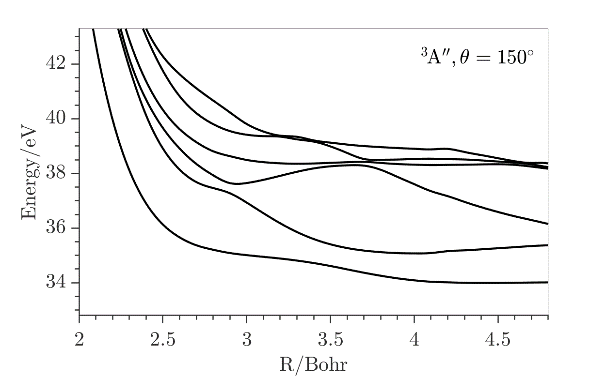

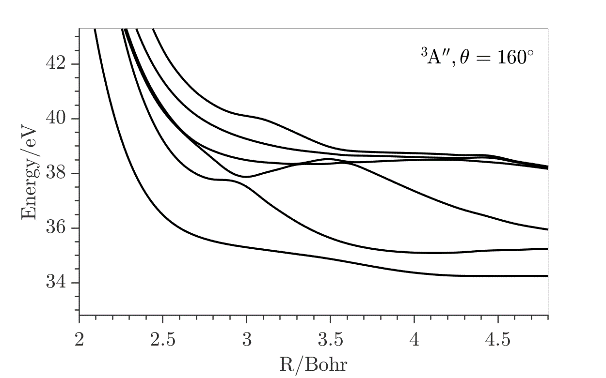

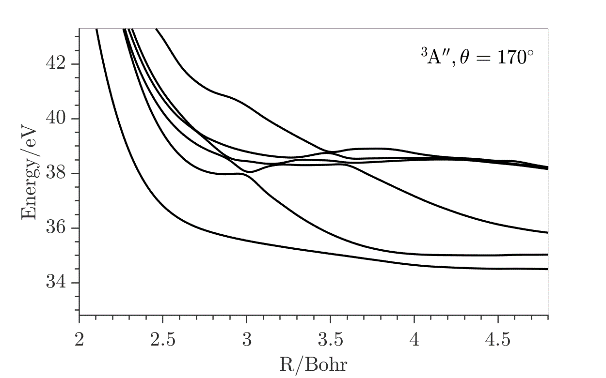

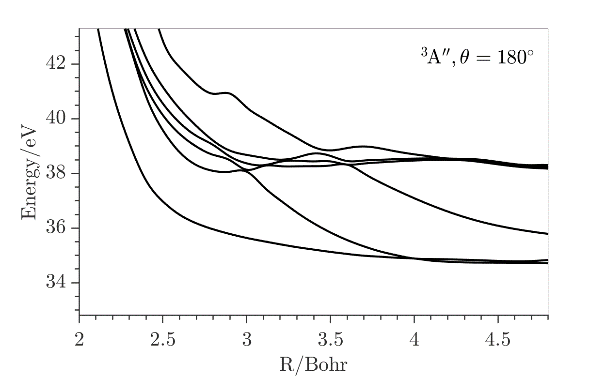


**Figure S6:** One dimensional cuts of the lowest ^3^A′′ states of SO_2_^2+^ for in-plane angle θ from 40° to 180° along the SO distance where the other SO is kept fixed at its value in SO_2_(X^1^A_1_) at equilibrium (i.e. 2.7 Bohr). The reference energy is that of SO_2_(X^1^A_1_) at equilibrium.

| 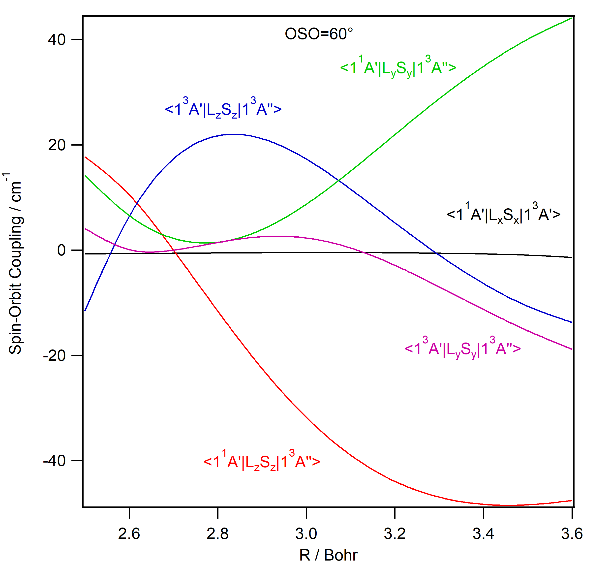 | 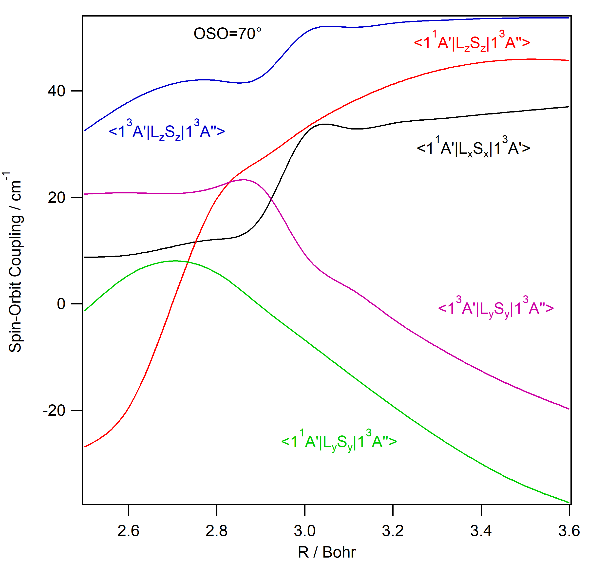 |
| --- | --- |
| 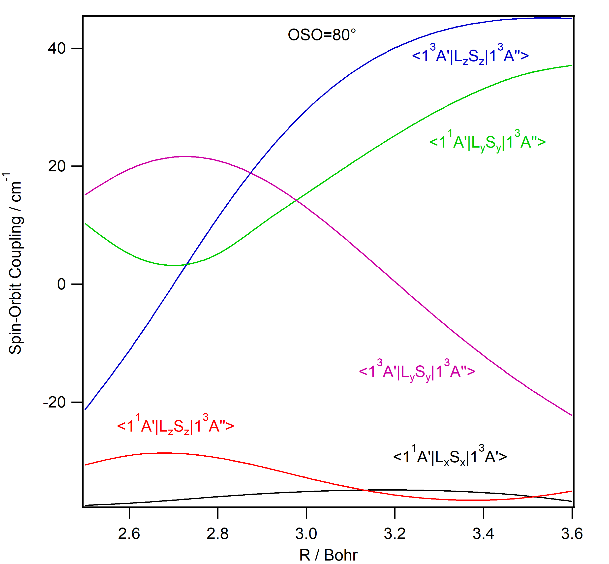 | 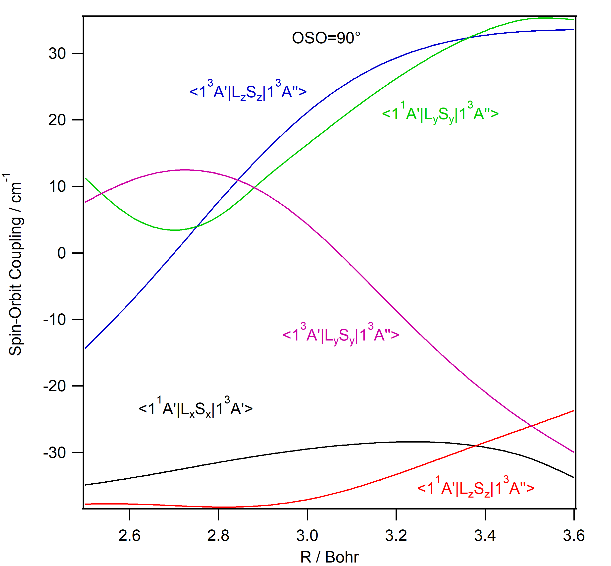 |
| 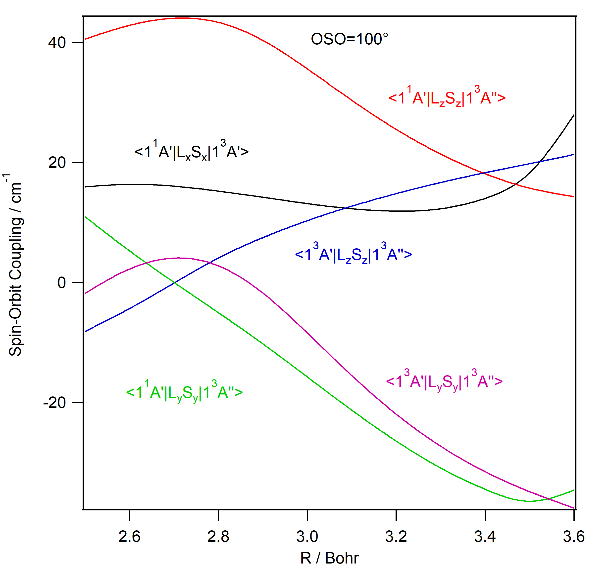 | 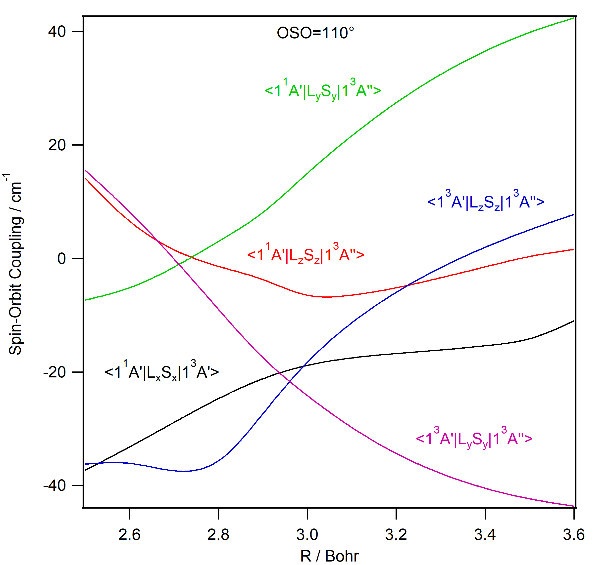 |

**Figure S7:** CASSCF/cc-pVTZ evolution of the non-vanishing spin-orbit integrals between the lowest ^1^A′, ^1^A′′, ^3^A′ and ^3^A′′ states of SO_2_^++^ along the SO distance for OSO in-plane angles = 60°, 70°, 80°, 90°, 100° and 110° where the other SO is kept fixed at its value in SO_2_(X^1^A_1_) at equilibrium (i.e. 2.7 Bohr).


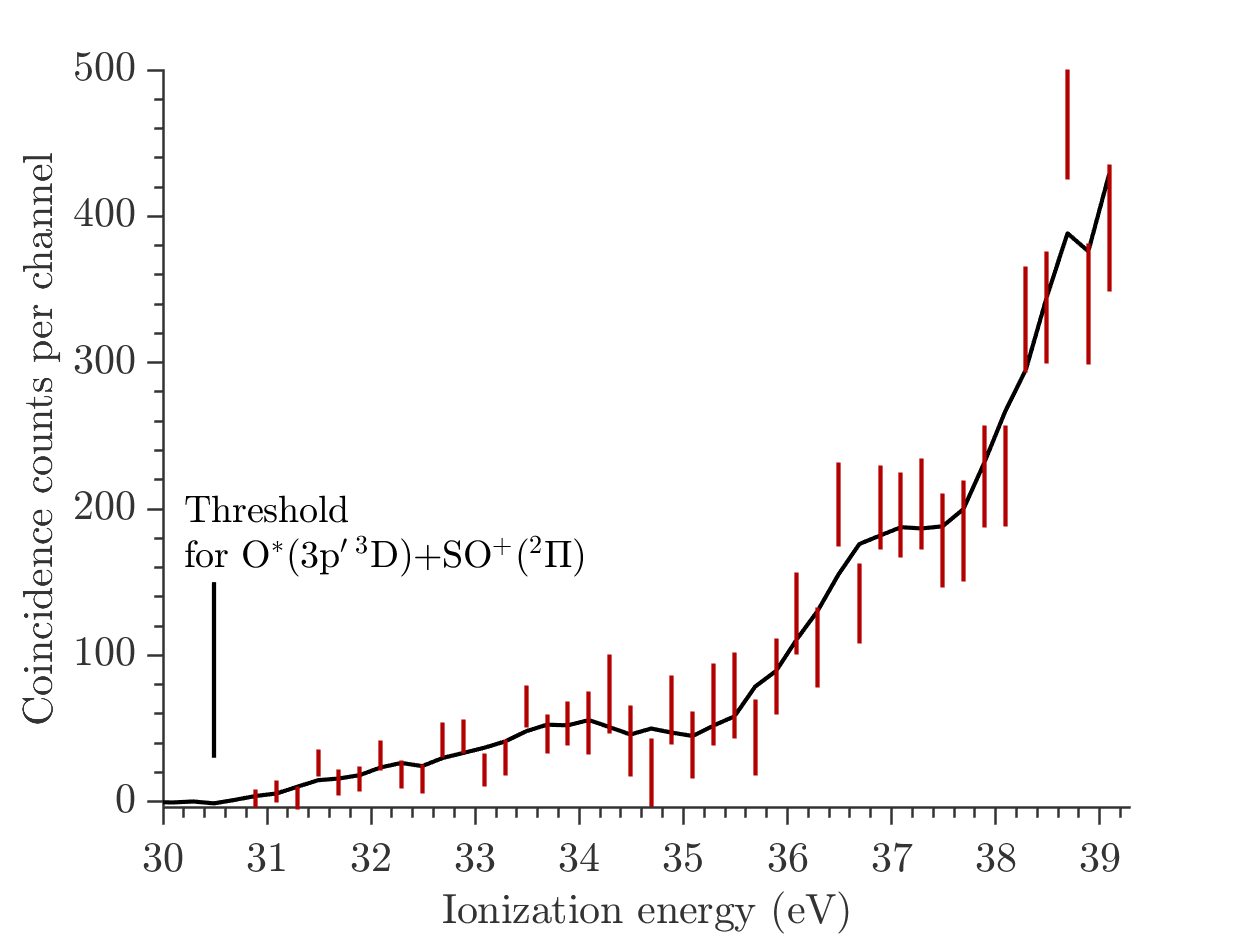


**Figure S8:** Yield spectrum for the O* (3p′ ^3^D) 🡪 O^+^ autoionizing states from photoionization of SO_2_ at 40.81 eV, giving electrons at near 0.5 eV over the whole range of ionization energies. Because this is a weak channel and strong underlying SO_2_^2+^ formation had to be subtracted, error bars are long and the smoothed curve is only indicative. This yield curve shows suggestions of structure reminiscent of the structure in the main double ionization spectrum. Because of the significant uncertainties (error bars), we should not place too much reliance on this apparent structure, but if the bump near 37.5 eV is to be believed, its existence is easily accounted for. The high-lying Rydberg states of SO_2_^+^* responsible lie at energies very close to those of the 2-h states on which they converge. Peaks in their density are therefore expected at energies indistinguishable at the current resolution from those of peaks in the parent double ionization spectrum.

**References**

1. 1. Ben Houria, A.; Ben Lakhdar, Z.; Hochlaf, M.; Kemp, F.; McNab, I. R. Theoretical investigation of the SO^2+^ dication and the photo-double ionization spectrum of SO. *J. Chem. Phys.* **2005,** *122*, 054303. [↑](#endnote-ref-1)
2. 2. Ben Houria, A.; Ben Lakhdar, Z.; Hochlaf, M. Spectroscopic and spin-orbit calculations on the SO^+^ radical cation. *J. Chem. Phys.* **2006,** *124*, 054313. [↑](#endnote-ref-2)
3. 3. <https://webbook.nist.gov> [↑](#endnote-ref-3)
